# Probiogenomic analysis of *Lactiplantibacillus plantarum* SPS109: A potential GABA-producing and cholesterol-lowering probiotic strain

**DOI:** 10.1016/j.heliyon.2024.e33823

**Published:** 2024-06-27

**Authors:** Nutwadee Chintakovid, Kamonnut Singkhamanan, Thunchanok Yaikhan, Natakorn Nokchan, Monwadee Wonglapsuwan, Jirayu Jitpakdee, Duangporn Kantachote, Komwit Surachat

**Affiliations:** a Department of Biomedical Sciences and Biomedical Engineering, Faculty of Medicine, Prince of Songkla University, Hat Yai, Songkhla, 90110, Thailand; b Division of Biological Science, Faculty of Science, Prince of Songkla University, Hat Yai, Songkhla, Thailand; c Translational Medicine Research Center, Faculty of Medicine, Prince of Songkla University, Hat Yai, Songkhla, 90110, Thailand

**Keywords:** Probiotic, *Choloylglycine hydrolase*, γ-aminobutyric acid, Genomic, Lactic acid bacteria

## Abstract

*Lactiplantibacillus plantarum* SPS109, an isolated strain of lactic acid bacteria (LAB) from fermented foods, showed remarkable potential as a probiotic with dual capabilities in γ-aminobutyric acid (GABA) production and cholesterol reduction. This study employs genomic and comparative analyses to search into the strain's genetic profile, safety features, and probiotic attributes. The safety assessment reveals the absence of virulence factors and antimicrobial resistance genes, while the genome uncovers bacteriocin-related elements, including sactipeptides and a cluster for putative plantaricins, strengthening its ability to combat diverse pathogens. Pangenome analysis revealed unique bacteriocin-related genes, specifically *lcnD* and *bcrA*, distinguishing SPS109 from four other *L. plantarum* strains producing GABA. In addition, genomic study emphasizes SPS109 strain distinctive features, two GABA-related genes responsible for GABA production and a bile tolerance gene (*cbh*) crucial for cholesterol reduction. Additionally, the analysis highlights several genes of potential probiotic properties, including stress tolerance, vitamin production, and antioxidant activity. In summary, *L. plantarum* SPS109 emerges as a promising probiotic candidate with versatile applications in the food and beverage industries, supported by its unique genomic features and safety profile.

## Highlights

• *L. plantarum* SPS109 shows dual functionality: GABA production & cholesterol reduction.
• Genomic analysis reveals unique features & probiotic attributes of SPS109.
• Safety assessment confirms absence of virulence factors & antimicrobial resistance genes.
• SPS109 exhibits potential as a probiotic with broad antimicrobial activity.
• The strain demonstrates stress tolerance, vitamin production & antioxidant activity.

## 1 Introduction

Lactic acid bacteria (LAB) are a remarkable group of microorganisms known for their ability to produce lactic acid through fermentation. They play a crucial role in numerous biological and industrial processes [1]. Most LAB species are harmless and are often designated as ‘Generally Recognized as Safe’ (GRAS) by the United States Food and Drug Administration (USFDA) and given the ‘Qualified Presumption of Safety’ (QPS) endorsement by the European Food Safety Authority (EFSA). To receive GRAS and QPS status, LAB must pass specific regulatory requirements. Due to this safety assurance, LAB are widely used in various food and beverage industries, with their GRAS and QPS status underscoring their significance [[2], [3], [4]]. Within the LAB group, one encounters a fascinating diversity of strains, including those belonging to the genera *Lactiplantibacillus*, *Pediococcus*, and *Streptococcus* [5]. Each species exhibits unique properties and functions, rendering them exceptionally well-suited for specific applications. Among the features of LAB are their distinctive metabolic characteristics, which encompass the production of organic acids, generation of unique flavors and aromas, and possession of proteolytic enzymes [1]. Remarkably, LAB strains are renowned for their probiotic properties, positioning them as indispensable players in the food industry, where they serve as functional starter cultures [2]. The versatile abilities of LAB extend their influence across a spectrum of industries, warranting a comprehensive exploration of their attributes and applications.

Numerous studies have shown that specific LAB strains, including those within the *Lactiplantibacillus* genus, are promising producers of gamma-aminobutyric acid (GABA) [[6], [7], [8], [9]]. GABA is synthesized through the decarboxylation of glutamic acid, catalyzed by the enzyme glutamate decarboxylase. This neurotransmitter has gained significant attention due to its well-documented physiological and pharmacological effects, which are associated with a range of health benefits. The potential advantages of GABA include its reported role in reducing anxiety and depression, boosting immune function, and regulating blood pressure, thereby potentially reducing the risk of cardiovascular issues [10]. Consequently, GABA-enriched fermented foods and beverages are emerging as potential trends in the realm of functional foods [11]. This highlights the vital role of LAB strains, particularly those capable of GABA production, in future trend of functional foods with the potential to enhance health and well-being.

In a previous study, *Lactiplantibacillus plantarum* SPS109 was isolated from fermented foods and demonstrated its capacity for GABA production [12]. Notably, when cultured in de Man, Rogosa, Sharpe (MRS) medium supplemented with 5 mg/ml monosodium glutamate (MSG), the SPS109 strain exhibited a remarkable 37.73 % increase in GABA content. In an initial screening process involving 139 LAB strains, the SPS109 strain emerged as a promising candidate for cholesterol degradation and subsequently advanced to the secondary screening stage [12]. However, it is still necessary to explore the genetic profile of this strain to highlight its phenotype and compare it with other strains.

This study aims to comprehensively analyze the genome of SPS109 to conduct a comprehensive genome analysis of the SPS109 strain. This analysis will help us better understand the genetic makeup of this strain and how it relates to its ability to produce GABA and its potential as a versatile probiotic. This genetic characterization will offer a deeper understanding of the strain's capabilities in this regard. Furthermore, we recognize the vital importance of safety when evaluating the deployment of microorganisms like SPS109 in the food industry. Therefore, we perform a thorough in silico safety assessment to support their phenotype. This assessment will ensure that the SPS109 strain poses no risks or concerns when employed in food-related applications. Essentially, our study aims to not only disclose the genetic insights of SPS109 but also establish its safety and assess its potential utility in the food industry.

## 2 Materials and methods

### 2.1 Genomic DNA extraction and sequencing

*L. plantarum* SPS109 was obtained from the previous study [12]. In brief, *L. plantarum* SPS109 was cultured in de Man, Rogosa, Sharpe medium (MRS) broth at 37 °C for 24 h under microaerobic conditions. For the stock culture, the MRS culture broth was mixed with 20 % glycerol and kept at −80 °C. The genomic DNA of *L. plantarum* SPS109 was then extracted and purified genomic DNA using the QIAamp® DNA Mini Kit (QIAGEN, Valencia, CA), following the manufacturer's instructions. DNA concentration and quality were quantified using a NanoDrop™ 2000/2000c spectrophotometer (Thermo Fisher Scientific, United States), and integrity and purity were assessed with agarose gel electrophoresis. The extracted DNA was then utilized for library preparation and sequenced using the BGISEQ-500 platform (BGI, China) to construct paired-end reads with a length of 150-bp.

### 2.2 Genome assembly and annotation

One Gbp of 150-bp paired-end were retrieved from the sequence provider. Subsequently, the raw sequence data underwent *de novo* assembled and annotated using BacSeq tools [13]. Functional annotation was performed by several tools and database including Rapid Annotations using Subsystems Technology (RAST) [14], and BLASTKOALA [15], for Kyoto Encyclopedia of Genes and Genomes (KEGG). Comparative analysis was visually represented using Proksee [16]. Evaluation of mobile genetic elements (MGEs), prophages, and antimicrobial resistance genes (ARGs) used mobileOG-db [17], Phigaro [18], VirulenceFinder [19,20] and the ResFinder web-based tool [21], respectively. In the search for ARGs, a 90 % threshold and a 60 % minimum length were applied as criteria. CRISPR (Clustered Regularly Interspaced Short Palindromic Repeats) arrays and their corresponding Cas proteins were pinpointed by employing CRISPRCasFinder [22]. The identification of ribosomally synthesized and posttranslationally modified peptides (RiPPs) and genes encoding bacteriocins was achieved through a sequence similarity search, and the findings were presented using the BAGEL4 webserver [23].

### 2.3 Comparative genomic and bioinformatics analysis

Four GABA-producing *L. plantarum* strains, namely DW12 [9], S11T3E, S2T10D [24], and CGMCC 1.243 [25], retrieved from the Reference Sequence (RefSeq) database, were selected for comparative analysis and pan-genome assessment alongside the SPS109. The selection criteria were established through a review of the literature and the availability of genomic sequences in the public database. Specifically, we chose the four strains based on the availability of their genomic data. Additionally, these strains were selected because they have published phenotypic and genomic data and are known for their ability to produce gamma-aminobutyric acid (GABA). To analyze the pan-genome of all five genomes, Roary [26] was utilized, applying a 95 % BLASTp threshold and standard parameters to identify core, accessory, and unique protein families. Multiple gene alignments and phylogenetic trees were then created using Geneious software [27] and the neighbor-joining method, with bootstrap testing conducted through 1000 repetitions to evaluate tree reliability. Additionally, a comparative analysis between *L. plantarum* SPS109 and the other four GABA-producing strains was executed. Proksee and BLAST were employed to visualize similarities in the coding sequences of these strains, while OrthoANI was used for average nucleotide identity analysis [28].

## 3 Results and discussions

### 3.1 Genome characteristics of *Lactiplantibacillus plantarum* SPS109 and species confirmation

**Fig. 1:**
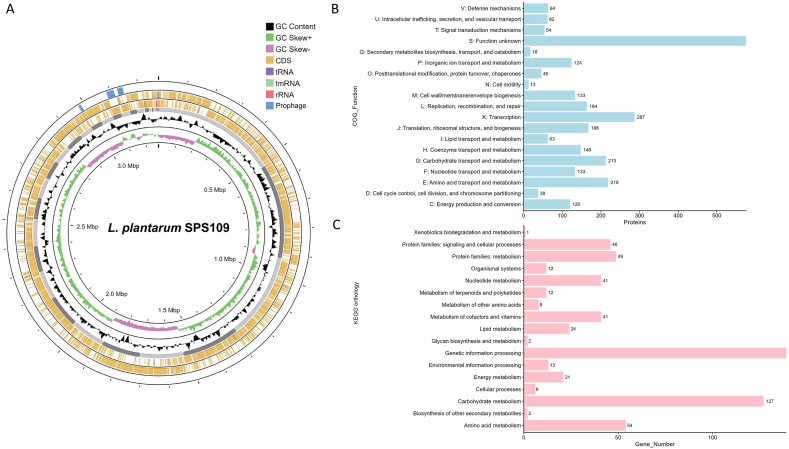
*L. plantarum* SPS109 circular genome and its function annotation. Moving from the outermost to the center: The first ring illustrates prophage regions (depicted in blue), while rings 2 and 3 display coding sequences (CDS), transfer RNA (tRNA), transfer-messenger RNA (tmRNA), and ribosomal RNA (rRNA) on both the forward and reverse strands (A). Cluster of Orthologous group (COG) functional (B) and KEGG orthology (C) categories of identified protein-coding genes in the *L. plantarum* SPS109 genome. (For interpretation of the references to colour in this figure legend, the reader is referred to the Web version of this article.)

The *L. plantarum* SPS109 draft genome is 3,269,561 bp long in 36 contigs with a GC content of 44.5 % (Table 1). No plasmid was found in the identification. The genome accession number is JAVKYM000000000. This genome contained 3063 coding sequences (CDS), 3 rRNA, 49 tRNA and 1 tmRNA. This table also presents the one CRISPRs, three Cas clusters, and one spacers (CRISPR/Cas) found in the chromosome (Table 1 and Fig. 1A). The number of CRISPRs, Cas clusters, and spacers in a bacterial genome refers to the components of the CRISPR-Cas system. These elements record previous infections, leaving a trace in the bacterial genome due to invasive genetic material. This record can influence the bacterial immune system's response to future infections [29]. The genomic features of the SPS109 genome are illustrated in Fig. 1A. The inner ring showed GC content and GC skew. Both forward and reverse strand CDS were shown in light brown colour ring. In these rings also presented the tRNA, tmRNA and rRNA. The outer ring presented three prophage regions. To confirm species, ANI value was calculated between the submitted genome and reference genome of *L. plantarum* SRCM100442 (Accession No. CP028221.1) from NCBI. The SPS109 strain showed high similarity with the reference genome (ANI = 98.92 %). In addition, the genome size and GC content (Table 1)showed similarity value when compared with other strains such as *L. plantarum* DW12 [9], S11T3E, S2T10D [24] and CGMCC 1.243 [25]. Therefore, this essential information ensures that SPS109 can be identified as *L. plantarum* based on its similar characteristics to other *L. plantarum* strain such as genome size, GC content, and ANI.

**Table 1:** Genome statistics of *L. plantarum* SPS109.

Features	Size
Genome size (bp)	3,269,561
Contigs	36
GC content (%)	44.5 %
Number of CDS	3063
tRNA	49
rRNA	3
tmRNA	1
CRISPR	1
Cas Cluster	3
Spacers (CRISPR/Cas)	1
Bacteriocin-liked encoding gene	2

### 3.2 Genome annotation and functional annotation

A total of 3063 coding sequences (CDS), 3 rRNA, 49 tRNA and 1 tmRNA were annotated in the SPS109 genome. From the predicted CDS, 1755 genes (57.29 %) were functionally annotated, and 1308 genes (42.70 %) were annotated as hypothetical protein. The 49 tRNA sequences correspond to 18 natural amino acids: Ala (2 sequences); Arg (5); Asn (3), Asp (3), Cys, Gln (3), Glu, Gly (4), His, Ile, Leu (6), Lys (2), Met (4), Phe, Pro (3), Ser (2), Thr (3), Trp (3) and Try (Supplementary Table 1).

The RAST server was utilized for functional annotation, offering a comprehensive overview of biological features. The subsystem coverage was 39 %, distributed across 328 SEED subsystems (Supplementary Table 2). The distribution of various functional groups revealed a prevalence of genes associated with general processes related to carbohydrates, amino acids and derivatives, protein metabolism, cofactors, vitamins, prosthetic groups, pigments, RNA metabolism, and nucleosides and nucleotides.

The examination of the COG functional groups categorized the 2927 genes (95.56 %) into 19 clusters (Fig. 1B). The largest proportion fell under the category of unknown function (S: 576 proteins). However, the characterization of these unidentified proteins was linked to hydrolase, membrane transport protein, and phage, affirming the distinctive and undiscovered potential of this strain. The remaining proteins were categorized under different functional groups; transcription (K:287); amino acid transport and metabolism (E: 219); replication, recombination and repair (L: 164); translation, ribosomal structure and biogenesis (J: 168); cell wall/membrane/envelope biogenesis (M: 133); inorganic ion transport and metabolism (P: 124); carbohydrate transport and metabolism (G: 213); nucleotide transport and metabolism (F: 133); coenzyme transport and metabolism (H: 148); intracellular trafficking, secretion, and vesicular transport (U: 62); signal transduction mechanisms (T: 54); defense mechanisms (V: 64); lipid transport and metabolism (I: 63); posttranslational modification, protein turnover, chaperones (O: 46); energy production and conversion (C: 120); cell cycle control, cell division, chromosome partitioning (D: 38); secondary metabolites biosynthesis, transport and catabolism (Q: 18), and cell motility (N: 13).

Furthermore, the KEGG functional annotation by BLASTKOALA assigned approximately 19.5 % of the genes (598 genes from 3063 genes) into 17 different functional categories (Fig. 1C), carbohydrate metabolism (127 genes), genetic information processing (139 genes), amino acid metabolism (54 genes) and etc.

### 3.3 Prophage and mobile genetic element analysis

Bacteriophages, commonly known as phages, are viruses that infect bacteria and recently gain attention due to the transferring of antibiotic-resistant strains in pathogenic bacteria. Phages occasionally offer advantages to their hosts by facilitating the other advantageous genes among different bacterial strains, however, phages can transfer of virulence factors into bacterial genome [18]. In the SPS109 strain, three prophage regions were detected as shown in Fig. 1A. The sizes of the regions were 52.7 kb, 40.4 kb, and 35.5 kb. All three-prophage taxonomy are *Siphoviridae* which is the most prevalent phage family infecting lactic acid bacteria (LAB) [30].

CRISPRs are genetic elements of repetitive DNA sequences, serving a crucial function in protecting the organism from foreign genetic elements [31]. In the *L. plantarum* SPS109 genome, one CRISPRs were found and identified with a length of 85 bp. The Cas3_Type I were found in 3 regions. CRISPR in LAB primarily functions as a defense mechanism against viral invasions, specifically bacteriophages. LAB incorporates small portions of viral DNA into their genomes through CRISPR arrays following past infections. This molecular memory allows the bacteria to recognize and cleave similar viral DNA upon re-infection, providing a form of acquired immunity against future viral threats [31,32]. MobileOG-db was preformed to identify mobile genetic element (MGE) in the SPS109 genome. The 143 MGEs were category into integration/excision (36), replication/recombination/repair (46), phage (42), stability/transfer/defense (10) and transfer (9). Bacterial MGE plays a crucial role in facilitating horizontal gene transfer (HGT), which involves the transfer of genetic material between nonparental bacterial lineages. MGEs are particularly problematic due to their pivotal role in the spreading of antibiotic resistance. The escalating global threat of antibiotic resistance poses a serious challenge to public health, allowing bacterial infections to persist through antibiotic treatment and diminishing the effectiveness of the drugs [17].

### 3.4 Safety assessments of probiotic

Since *L. plantarum* SPS109 is a potential probiotic that could be a producer for several dietary products. Therefore, assessing the risk of antimicrobial resistance ability and MGEs in both phenotype and genotype is important for consumers and also to prevent the distribution of antimicrobial resistance genes in the human gut environment. The safety assessments including virulence gene, AMR and bacteriocin were investigated.

#### 3.4.1 Virulence and AMR

No virulence genes were detected using the VirulenceFinder tool. However, RAST annotation revealed 46 genes associated with defense mechanisms in the SPS109. This discrepancy raises an intriguing question about the relationship between these findings. One possible explanation could be the limitations of the VirulenceFinder tool, which might not capture all types of virulence factors in all bacterial strains but only common ones. Therefore, the integration of virulent genes from both sources could provide us with more comprehensive information. Due to the gene identified as defense mechanisms could play dual roles. While these genes are typically recognized as virulence factors in pathogens, aiding in their survival in the host environment under physiological stresses, they could also contribute to the survival of a probiotic in the gut [33]. Regarding safety concerns, an absence of detected genes associated with virulence factors in strain SPS109 suggests a lack of harm to humans, confirming the non-existence of virulence genes.

No antimicrobial resistance (AMR) gene was found in the ResFinder database. This assurance of safety was consistent with the earlier findings. The antibiotics susceptibility test revealed that SPS109 was susceptible to various antibiotics, including chloramphenicol, kanamycin, penicillin, and others [34]. We further identified mobile genetic elements (MGEs) in strain SPS109 using the MEGFinder database. Four insertion sequences (ISs) were detected, with no genes encoded. This could suggest a lack of ability for transmission.

#### 3.4.2 Bacteriocin identification

**Fig. 2:**
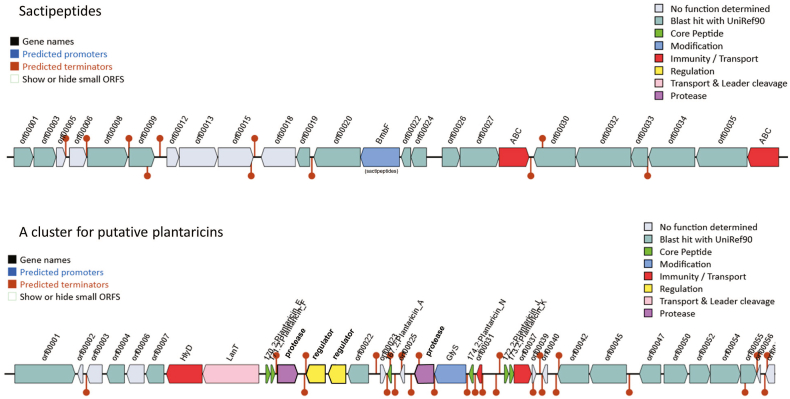
The L. *plantarum* SPS109 genome analysis of bacteriocin genes arrangement using the BAGEL4. The area of interest (AOI) at node2 and 6 indicates sactipeptides and a cluster of PlnA, PlnE, PlnF, PlnJ, PlnK, and PlnN, respectively.

The possibility of using bacteriocins and its applications to reduce microbiological spoilage and preserve food has recently been recognized worldwide [35]. *In silico* bacteriocin identification of the *L. plantarum* SPS109 sequences revealed that two bacteriocin-encoding genes were located in two different Areas of Interest (AOI). The first one is in contig 2 (starting from 67,223 to 87,223), which contains genes related to sactipeptides. The second one is in contig 6 (starting from 54,974 to 84,472), harboring a cluster for putative plantaricins (Fig. 2).

Sactipeptides belong to ribosomally synthesized and post-translationally modified peptides (RiPPs) family. Under the catalysis of sactisynthases, the thioether pattern in sactipeptides provides improved structural, thermal, and proteolytic stability which makes them attractive scaffolds for the development of novel biotherapeutics. In addition, certain sactipeptides exhibit a narrow antibiotic efficacy against drug-resistant bacteria. This could be crucial in the future, given the imminent threat of a significant antibiotic crisis worldwide [36].

The *in-silico* analysis of the *L. plantarum* SPS109 genome revealed six types of plantaricin (PInA, PInE, PInF, PInJ, PInK, and PInN) as illustrated in Fig. 2. These plantaricins have been reported to exhibit robust protective properties against urinary tract infection (UTI) [37]. Moreover, one of the plantaricins demonstrated effective inhibition of the growth of *Listeria monocytogenes* and *Staphylococcus aureus* [38]. Plantaricin A (PlnA) enhances the effectiveness of antibiotics by augmenting the permeability of the bacterial outer membrane [39]. These peptides are initially synthesized as precursors and subsequently induce the activation of genes that encode two peptide bacteriocins, Plantaricin E/F and J/K [40]. Additionally, the plantaricin locus of SPS109 includes genes that encode LanT (bacteriocin ABC-transporter, ATP-binding, and permease protein PlnG). The antibacterial activity of SPS109 was performed under unadjusted pH (3.50) culture supernatants showed inhibition zone against several pathogens including *Bacillus cereus* TISTR 687, *Escherichia coli* ATCC 25922, *L. monocytogenes* DMST 17303, *Salmonella* Typhi DMST 22842, *S. aureus* ATCC 25923, and *Streptococcus mutans* ATCC 25175 [34].

### 3.5 Probiotic marker genes

LAB strains are well-known for their probiotic attributes which including stress tolerance, antioxidant, and immunomodulatory activities, making them essential contributors to the food industry [2]. In addition, probiotics are selected based on unique characteristics, including distinct health advantages, the ability to survive and persist in the host, demonstrated safety, and stability [41]. For optimal health benefits, probiotics should demonstrate positive effects on human health, such as reducing cholesterol, producing GABA, enhancing the immune system, or promoting digestive health. Additionally, potential probiotic strains should exhibit resilience in surviving the harsh conditions of the digestive system and persist in the gastrointestinal tract. Another crucial characteristic is safety for human consumption, ensuring the absence of toxicity, allergens, and pathogenic traits. It is equally important that probiotics maintain their viability and effectiveness throughout the entire production process, storage, and the entire shelf life of the product.

Based on published literature data, they reveal probiotic marker genes (PMG) which related to their characteristics and commonly found in all strains. The proposed genes could be a strain-specific peculiarity in their probiotic potential [3,42], the SPS109 strains revealed various probiotic marker genes (as listed in Table 2). This finding indicated its probiotic functions at genomic levels. This also supports the idea that *L. plantarum* SPS109 could be a promising candidate probiotic for use in the food industry.

**Table 2:** Probiotics marker genes (PMGs) identified specifically in the SPS109 strain. These genes were compared to the PMGs listed in Carpi et al., 2022 and Kandasamy et al., 2022. The genes found in the SPS109 genome that match the PMGs lists indicate its potential as a probiotic.

Gene	Function
Heat stress	
*htpX*	Heat shock protein htpx
*hrcA*	Heat-inducible transcriptional repressor
*hslO*	Molecular chaperone Hsp33
*dnaK*	HSPA9; molecular chaperone DnaK
*dnaJ*	Molecular chaperone dnaj
*ctsR*	Transcriptional regulator of stress and heat shock response
*grpE*	Molecular chaperone GrpE
*groS*	10 kDa chaperonin
*clpB*	ATP-dependent Clp protease ATP-binding subunit ClpB
*clpE*	ATP-dependent Clp protease ATP-binding subunit ClpE
*clpX*	ATP-dependent Clp protease ATP-binding subunit ClpX
*clpP_1*	ATP-dependent Clp protease proteolytic subunit
*hslV*	ATP-dependent HslUV protease, peptidase subunit HslV
*htrA*	Serine protease HtrA
**Osmotic stress**	
*gbuB*	Glycine betaine/carnitine transport permease protein GbuB
*glpF_1*	Glycerol uptake facilitator
*glpF_2*	Glycerol uptake facilitator
*opuCB*	Glycine betaine/carnitine/choline transport system permease protein OpuCB
**Acid stress**	
*atpA*	F-type H+/Na + -transporting ATPase subunit alpha
*atpH*	F-type H + -transporting ATPase subunit delta
*atpG*	F-type H + -transporting ATPase subunit gamma
*atpD*	F-type H+/Na + -transporting ATPase subunit beta
*atpC*	F-type H + -transporting ATPase subunit epsilon
*gadB*	gadB, gadA, GAD; glutamate decarboxylase
*copA*	Copper transporting ATPase
*pgk*	Phosphoglycerate kinase
*gpmB*	Phosphoglycerate mutase GpmB
*pspA_1*	Phosphoserine phosphatase 1
*guaA*	GMP synthase [glutamine-hydrolyzing]
*ldh_1*	l-lactate dehydrogenase 1
*plsC*	1-acylglycerol-3-phosphate O-acyltransferase
*pyk*	Pyruvate kinase
*recA*	Protein RecA
*relA*	GTP pyrophosphokinase
*uvrA/uvrA_1*	uvrABC system protein A
*yjbM*	GTP pyrophosphokinase YjbM
*ywaC*	GTP pyrophosphokinase YwaC
**Bile tolerance**	
*arcB*	Ornithine carbamoyltransferase
*argS*	Arginine--tRNA ligase
*cbh*	Choloylglycine hydrolase
*ppaC*	Manganese-dependent inorganic pyrophosphatase
*pgk*	Phosphoglycerate kinase
*dps*	DNA protection during starvation protein
*glnA*	Glutamine synthetase
*nagB*	Glucosamine-6-phosphate deaminase
*oppA_1*	Oligopeptide–binding protein OppA
*oppA_2*	Oligopeptide–binding protein OppA
*pdhD_2*	Dihydrolipoyl dehydrogenase
*pepO*	Endopeptidase PepO
*ponA*	Penicillin-binding protein 1 A
*pyrG*	CTP synthase
*rplD*	50 S ribosomal protein L4
*rplE*	50 S ribosomal protein L5
*rplF*	50 S ribosomal protein L6
*rpsC*	30 S ribosomal protein S3
*rpsE*	30 S ribosomal protein S5
*luxS*	S-ribosylhomocysteine lyase
**Adhesion**	
*lspA*	Lipoprotein signal peptidase II
*tuf*	Elongation factor Tu
*tpiA*	Triosephosphate isomerase (TIM)
*pgaC*	Poly-beta-1,6-*N*-acetyl-d-glucosamine synthase
*eno*	Enolase
*eno2*	Enolase 2
*pgi*	Glucose-6-phosphate isomerase
*epsH*	Putative glycosyltransferase epsh
*exoA*	Exodeoxyribonuclease III
*pycA*	Pyruvate carboxylase
**Antioxidant**	
*nrdH*	Glutaredoxin
*tpx*	Thiol peroxidase
*trxB*	Thioredoxin reductase
*msrB*	Peptide-methionine (R)-S-oxide reductase
*msrC*	l-methionine (R)-S-oxide reductase
**Immunomodulation**	
*dltA*	d-alanine—poly(phosphoribitol) ligase subunit 1
*dltD*	d-alanine transfer protein
**Gut persistence**	
*celB*	PTS system, cellobiose-specific EIIC component
*treA*	Trehalose-6-phosphate hydrolase

### 3.6 A promising probiotic, GABA producer and lowering cholesterol

GAD is the essential enzyme in GABA biosynthesis. The conversion of glutamate to GABA by GAD is the first step of the gamma-aminobutyrate (GABA) shunt pathway. The existing of *glutamate decarboxylase* (*gadB*) led to a higher GABA production [43]. The genome of the SPS109 contains one *gadB* gene. Thus, this gene is one of the others that responsible for inducing the GABA production of SPS109 and facilitate a GABA production which cause 37.73 % [12]. The second enzyme involved in the GABA pathway is succinate semialdehyde dehydrogenase (SSADH), which converts succinate semialdehyde (SSA) to succinate, returning it to the TCA cycle. However, SSADH functions primarily as a degradation pathway rather than a synthesis pathway for GABA [44]. The SSADH encodes by two paralogous *gabD1* and *gabD2* [45]. The succinate-semialdehyde dehydrogenase NADP^+^ (*gabD1*) was also found in the SPS109 genome.

Recently, bile salt hydrolase (BSH, EC 3.5.1.24) activity of probiotic has been recognized as a pivotal factor contributing to cholesterol-lowering effects. BSH can catalyze the hydrolysis of conjugated bile salts into deconjugated bile acids (BAs) become less soluble and are excreted in feces instead leading to cholesterol-lowering [46]. In addition, BSH facilitates growth and colonization of bacterial [47]. The responsible enzymes are collectively called BSH and belong to the choloylglycine hydrolase family [48] and BSH which alternatively names as *choloylglycine hydrolase* gene (*cbh*) encode choloylglycine hydrolase [46]. Unexpectedly, the SPS109 genome contained the *cbh* gene correlated with their phenotype. The SPS109 was initially screened by cholesterol degradation and GABA-producing. The SPS109 abled to reduced cholesterol content in the culture medium 10.89 % after 48 h of incubation [12].

### 3.7 Comparative and pan-genome analysis of 5 *L. plantarum* GABA producing strains

**Fig. 3:**
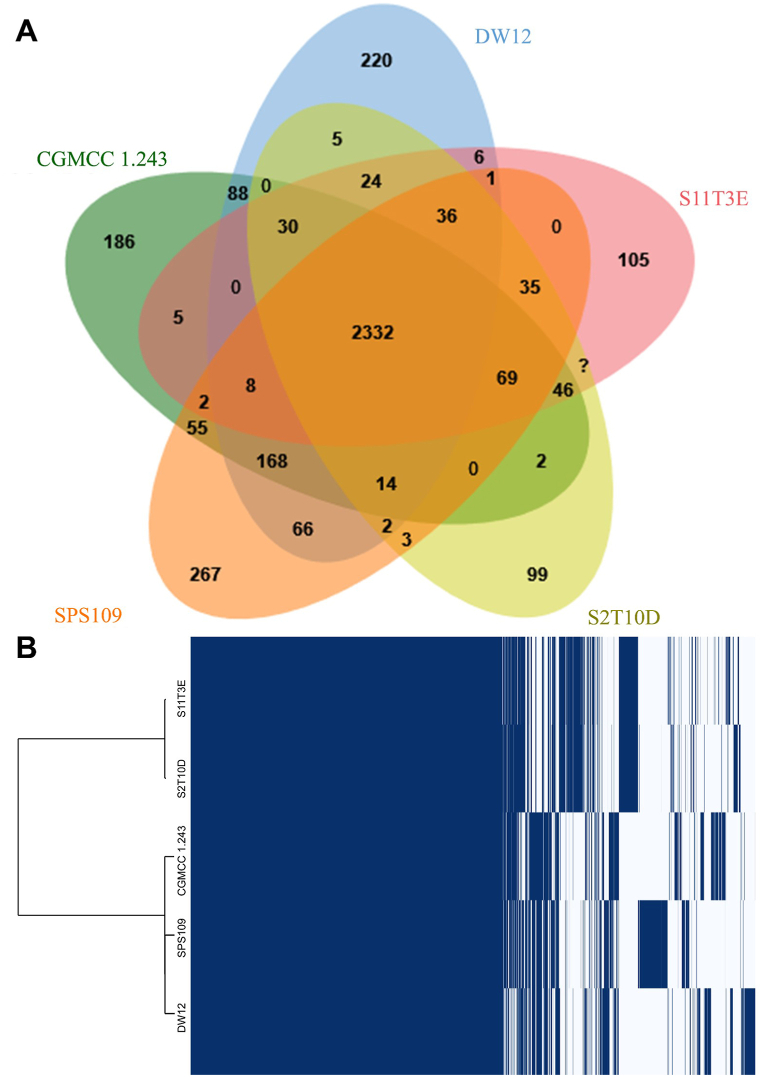
Comparative analysis of genes annotated among *L. plantarum* SPS109, DW12, S11T3E, S2T10D and CGMCC 1.243. Venn diagram showed different number of unique and sharing genes among the *L. plantarum* genome (A). Pan-genome presented a core and accessory gene by comparing with the genomes of *L. plantarum* (B).

A comparative genomic and pan-genome analysis was conducted to assess the diversity of genes within *L. plantarum* strains. This involved a comparison with the genomes of *L. plantarum* DW12 [9], S11T3E, S2T10D [24] and CGMCC 1.243 [25] (Fig. 3A). All the 5 strains of *L. plantarum* shared 2332 genes. Genes that are found only in each strain from *L. plantarum* DW12, S11T3E, S2T10D and CGMCC 1.243 are 220, 105, 99 and 186, respectively. Most of the unique genes are hypothetical proteins. The number of functional annotated genes are 52 genes from DW12 strain, 44 genes from S11T3E strain, 37 genes from S2T10D strain and 42 genes from CGMCC 1.243 strain (Supplementary Table 3). The *gadB* presented in all 5 genomes. However, some of the essential genes were found in some strains. Gene that related to lowering cholesterol and glycerol uptake facilitator (*glpF_1*) are found in only *L. plantarum* SPS109, DW12 and CGMCC 1.243.

By comparing with the other strains, the SPS109 strain was identified 267 unique genes which had a highest number. Of the 267 genes, 89 genes were functionally annotated. The eighty-nine functionally annotated genes were listed in Table 3. Several interesting genes are discussed below. Gycine betaine/carnitine transport permease protein encoded by *gbuA*, *gbuB* and *gbuC* related to osmotic stress was found in the SPS109 genome. *Glycine betaine porter II* (GbuB) is a class of transport systems that was identified as an osmotically activated and cold-activated glycine betaine ABC transporter [49]. This protein enhances the survival rate of bacteria under the osmotic stress by stimulating an accumulation of the glycine betaine [42,49]. These three genes might help the SPS109 survive in different conditions of osmotic stress.

**Table 3:** The unique genes specific to *L. plantarum* SPS109 through pan-genome comparison. These unique genes were present only in the SPS109 strain and absent in other strains.

Gene	Annotation
*gpmA_2*	2,3-bisphosphoglycerate-dependent phosphoglycerate mutase
*COQ5*	2-methoxy-6-polyprenyl-1,4-benzoquinol methylase, mitochondrial
*dinG_3*	3′-5′ exonuclease DinG
*otnC*	3-oxo-tetronate 4-phosphate decarboxylase
*sasA_4*	Adaptive-response sensory-kinase SasA
*yveA*	Aspartate-proton symporter
*atpB*	ATP synthase subunit a
*atpF*	ATP synthase subunit b
*atpE*	ATP synthase subunit c
*bcrA_2*	Bacitracin transport ATP-binding protein BcrA
*ccpA_2*	Catabolite control protein A
*smc_6*	Chromosome partition protein Smc
*smc_9*	Chromosome partition protein Smc
*alsE*	d-allulose-6-phosphate 3-epimerase
*dap*	d-aminopeptidase
*bacC_2*	Dihydroanticapsin 7-dehydrogenase
*mshA*	D-inositol-3-phosphate glycosyltransferase
*topB*	DNA topoisomerase 3
*tag_3*	DNA-3-methyladenine glycosylase 1
*fsaA*	Fructose-6-phosphate aldolase 1
*gbuA*	Glycine betaine/carnitine transport ATP-binding protein GbuA
*gbuC*	Glycine betaine/carnitine transport binding protein GbuC
*gbuB*	Glycine betaine/carnitine transport permease protein GbuB
*clcA_2*	H(+)/Cl(−) exchange transporter ClcA
*hdfR_4*	HTH-type transcriptional regulator HdfR
*ydiM_1*	Inner membrane transport protein YdiM
*iolG_3*	Inositol 2-dehydrogenase
*iolE*	Inosose dehydratase
*lcnD_2*	Lactococcin A secretion protein LcnD
*mscL_2*	Large-conductance mechanosensitive channel
*levS*	Levansucrase
*kup_3*	Low affinity potassium transport system protein kup
*tdh_3*	l-threonine 3-dehydrogenase
*iolT_1*	Major myo-inositol transporter IolT
*iolT_2*	Major myo-inositol transporter IolT
*iolG_1*	Myo-inositol 2-dehydrogenase
*iolG_2*	Myo-inositol 2-dehydrogenase
*iolG_4*	Myo-inositol 2-dehydrogenase
*napA_3*	Na(+)/H(+) antiporter
*tagA_1*	*N*-acetylglucosaminyldiphosphoundecaprenol *N*-acetyl-beta-d-mannosaminyltransferase
*malL_4*	Oligo-1,6-glucosidase
*oppD_1*	Oligopeptide transport ATP-binding protein OppD
*oppF_1*	Oligopeptide transport ATP-binding protein OppF
*oppC*	Oligopeptide transport system permease protein OppC
*sarA*	Oligopeptide-binding protein SarA
–	Protein ADP-ribosyltransferase
*frwD*	PTS system fructose-like EIIB component 3
*fruA_1*	PTS system fructose-specific EIIB'BC component
*srlB_2*	PTS system glucitol/sorbitol-specific EIIA component
*srlE_2*	PTS system glucitol/sorbitol-specific EIIB component
*srlA_2*	PTS system glucitol/sorbitol-specific EIIC component
*manP_1*	PTS system mannose-specific EIIBCA component
*epsF*	Putative glycosyltransferase EpsF
–	Putative HTH-type transcriptional regulator
*licR_2*	Putative licABCH operon regulator
*yfcA_1*	Putative membrane transporter protein YfcA
*ycnE_2*	Putative monooxygenase YcnE
–	Putative NrdI-like protein
*rfbX*	Putative O-antigen transporter
*yedJ_2*	Putative protein YedJ
*bin3_1*	Putative transposon Tn552 DNA-invertase bin3
*bin3_2*	Putative transposon Tn552 DNA-invertase bin3
*rihA_1*	Pyrimidine-specific ribonucleoside hydrolase RihA
*ribBA_2*	Riboflavin biosynthesis protein RibBA
*nrdE*	Ribonucleoside-diphosphate reductase 2 subunit alpha
*nrdF*	Ribonucleoside-diphosphate reductase 2 subunit beta
*nrdF2_2*	Ribonucleoside-diphosphate reductase subunit beta nrdF2
*regX3*	Sensory transduction protein regX3
*sotB*	Sugar efflux transporter
*manR*	Transcriptional regulator ManR
*tkt_2*	Transketolase
*xerC_3*	Tyrosine recombinase XerC
*xerC_4*	Tyrosine recombinase XerC
*ptk*	Tyrosine-protein kinase ptk
*mnaA*	UDP-*N*-acetylglucosamine 2-epimerase
*urdA_3*	Urocanate reductase
*btuD_4*	Vitamin B12 import ATP-binding protein BtuD

A study from Jitpakdee et al. reported an antibiotic activity of SPS109 and it was susceptible to several antibiotics such as carbenicillin, chloramphenicol, and kanamycin and able to inhibit the pathogen microorganism under pH unadjusted condition (pH = 3.50) [34]. This is also consistent with the genome annotation result. Lactococcin A secretion protein encoded by *lcnD* was found in the genome indicating a promising role of producing the bacteriocin in this strain. The *lcnD* stimulates the Lactococcin A (LcnA) expression led to bacteriocin production from *Lactococcus lactis* inducing the membrane leakage and cell death [50,51]. Bacteriocin transport ATP-binding protein BcrA (*bcrA_2*) and was also found in the SPS109.

An ABC transporter facilitates the uptake of vitamin B12, and this process is associated with the BtuCDF protein, which is responsible for energy coupling to the transport system. The transporter complex consists of two ATP-binding cassettes (BtuD) and two membrane-spanning subunits (BtuC), collaborating with BtuF, a substrate-binding protein that carries vitamin B12. Only SPS109 genome presented *btuD* gene encoding Vitamin B12 import ATP-binding protein BtuD [52,53]. An analysis of the genomes of *Lactobacillus acidophilus* isolates revealed a single nucleotide polymorphism (SNP) in the BtuD region, resulting in the inactivation of the transport system for utilizing vitamin B12 [52]. Another gene, *ribBA_2* involves biosynthesis of B2 was also uniquely found in SPS109.

A functional proteolytic system is essential for obtaining the necessary amino acids, with one of its key components being the Oligopeptide transport system (Opp). The Opp system, categorized within the ATP-binding cassette (ABC) superfamily, functions as an ATP-driven oligopeptide transporter. It comprises five proteins: two transmembrane proteins (OppB and OppC), two ATP-binding proteins (OppD and OppF), and the substrate binding protein (OppA). In the genome of SPS109, three genes (*oppD*, *oppF*, and *oppC*) were identified, responsible for encoding the ATP-binding protein in the oligopeptide transport system.

Pangenome analysis of the *L. plantarum* pan-genome revealed the presence of 886 core genes, 494 soft core genes, 2686 shell genes, and 31,908 cloud genes, among a total of 35,969 genes (Fig. 3B). The considerable presence of cloud genes indicates a notable diversity among the 941 studied strains of *L. plantarum*, highlighting the fundamentally ‘open’ characteristic of the *L. plantarum* pan-genome. The number of core genes identified in this study was found to be lower than in a previous investigation [42], which reported 1436 core genes across 127 complete genomes of *L. plantarum*. The reduction in the number of core genome genes as more genomes are added suggests a pattern in the evolution and diversity of the species. Early in the analysis, the identification of core genes highlights the foundational genetic elements crucial for the species. As additional genomes are integrated, the diminishing number of new core genes suggests a stabilization in the essential genetic components, indicating a saturation point in the species' core genome. This observed trend may provide insights into the evolutionary dynamics and genetic diversity of the studied organisms, suggesting that the core genome reaches a relatively stable state as more genomes are considered in the analysis.

While no AMR gene was found in ResFinder database, but the Roary analysis (Fig. 3B) revealed that 11 AMR genes which related to chloramphenicol acetyltransferase (*cat*), penicillin-binding protein (*pbpF*, *penA*, *ponA*, *pbpB*, *pbpX_1*, *pbpX_2* and *pbpF*) and tetracycline resistance protein (*tetO* and *tetA*). Even though the genes related to AMR were found in the SPS109 genome but the SPS109 strain was susceptible to penicillin and tetracycline according to the previous study [34]. Additionally, this strain was confirmed to be safe for producing a functional fermented whey beverage [34]. The observation of chloramphenicol gene (*cat*) was consecutive found in several *Lactobacillus* spp. Genes related to chloramphenicol (*cat*) and tetracycline (*tetM*, *tetS*, *tet(W)*, *tet(O)* and *tetQ*) are also commonly found in *Lactobacillus* spp. [54].

## 4 Conclusions

The *in-silico* analysis of *L. plantarum* SPS109 provides information of genome characteristics, functional annotation, safety assessment, and probiotic properties. The SPS109 strain was identified as a high GABA-producing strain with cholesterol-lowering capabilities [12]. The genome analysis supports these phenotypes by revealing the presence of GABA-related gene and *cbh* gene that play a crucial role in lowering cholesterol. Safety assessment analyses confirm its safety profile which has no AMR genes and highlight potential probiotic properties, including stress tolerance, vitamin production, and antioxidant activity. Consequently, *L. plantarum* SPS109 emerges as a promising probiotic candidate suitable for applications in the food and beverage industries. Foods incorporating L. *plantarum* SPS109 could potentially contribute to promoting relaxation and reducing stress, as GABA is known for its calming effects on the nervous system. In addition, products containing *L. plantarum* SPS109 may be beneficial for individuals aiming to manage their cholesterol levels which can promote heart health. The development of products containing *L. plantarum* SPS109 could lead to natural added products with unique health-promoting features, ranging from high GABA content, cholesterol management to and overall well-being. In sum, foods fermented with *L. plantarum* SPS109 have the potential to serve as potent natural sources of GABA and compounds associated with cholesterol reduction. Future studies should focus on the scaled-up production of *L. plantarum* SPS109 as a starter culture in the fermentation of milk and other food products. It is crucial to examine the specific mechanisms underlying GABA production and the identification of compounds responsible for cholesterol reduction in commercial applications.

## Funding

This research was supported by National Science, Research and Innovation Fund (NSRF) and 10.13039/501100004508Prince of Songkla University (Ref. No. MED6701023S). In addition, this research was supported by the Postdoctoral Fellowship from 10.13039/501100004508Prince of Songkla University, Thailand, and this research has also received funding support from the NSRF via the Program Management Unit for Human Resources & Institutional Development, Research and Innovation, grant number B13F660074.

## Data availability statement

The genome is accessible through BioProject number PRJNA1014225, with corresponding BioSample number SAMN37321373.

## CRediT authorship contribution statement

**Nutwadee Chintakovid:** Writing – review & editing, Writing – original draft, Validation, Software, Methodology, Investigation. **Kamonnut Singkhamanan:** Writing – review & editing, Writing – original draft, Project administration, Methodology, Investigation, Conceptualization. **Thunchanok Yaikhan:** Methodology, Investigation, Formal analysis, Data curation. **Natakorn Nokchan:** Methodology, Formal analysis, Data curation. **Monwadee Wonglapsuwan:** Writing – review & editing, Methodology, Investigation, Funding acquisition, Conceptualization. **Jirayu Jitpakdee:** Resources, Methodology. **Duangporn Kantachote:** Writing – review & editing, Supervision, Resources. **Komwit Surachat:** Writing – review & editing, Writing – original draft, Validation, Supervision, Software, Resources, Project administration, Methodology, Investigation, Funding acquisition, Conceptualization.

## Declaration of competing interest

The authors declare that they have no known competing financial interests or personal relationships that could have appeared to influence the work reported in this paper.
